# Allegheny County Medical Society

**Published:** 1888-10

**Authors:** W. M. Brinton

**Affiliations:** President, in the Chair


### ALLEGHENY COUNTY MEDICAL SOCIETY.

### SPECIAL MEETING, AUGUST 21ST, 1888.

W. M. BRINTON, M. D., PRESIDENT, IN THE CHAIR.

Dr. T. W. Shaw read the report of committee on the death of Dr. Thomas J. Gallaher:

It is with feelings of profound sorrow that we record the death of Dr. Thomas J. Gallaher, one of our oldest and most efficient members. The death of such a one requires more than a mere passing notice from the members of this Society. He was one of the founders of this organization, and one of its most active and useful members. He was rarely absent from its meetings, and was always seeking to advance its usefulness.

It was my privilege to know Dr. Gallaher intimately, both professionally and socially, for more than forty years. We were students of medicine at the same time, and graduated in the same class from the Medical Department of the University of Pennsylvania. As a student, he was a model of industry and labor. After receiving his diploma, he entered upon practice with an energy and a devotion that few men manifest. He labored under many and great disadvantages. He was poor, had few friends, and could bring to his aid but little influence to establish him in his profession. He commenced his career at the very bottom, and fought his way to an honorable and distinguished prominence. Dr. Gallaher had a large practice. He enjoyed to an unusual degree the confidence and love of his patients, and his professional brethren admired his candid and sincere nature. Throughout his long and busy practice he scorned to do a mean or unprofessional act. He was a man of pure life, of kindly feelings, and of pleasant and agreeable manners; and whether calling as a friend or as a physician, he was always welcome to the homes of the rich and the dwellings of the poor.

He had been in failing health for two years past, but his most intimate friends noticed a decline of his health and his spirits about four years ago. For the last year he has been almost helpless. Last September, when the International Medical Congress met in Washington, he went there expecting to take part, and hoping to renew the friendships that he had made at Copenhagen. But after reaching his hotel, he was unable to leave his room, and he was brought home to the bed from which he never rose.

He has fallen in the great battle of life. He goes down to the grave sincerely mourned, but leaves an enviable record and a good name behind him. As the friend of his earlier manhood, and a companion and professional brother of his riper years, I wish to bear this tribute to his memory:

*Resolved*, That this report be entered upon the minutes of the Society, and a copy sent to the family of the deceased.

On motion, the report of the committee was adopted, and the Secretary ordered to spread the same on the minutes of the Society, and to send a copy thereof to the family of the deceased.

### FATAL CASE OF TYPHOID FEVER.

Dr. J. C. McMullen reported a case of typhoid fever. The case proceeded favorably until upon the seventh day milk was given the patient. This was followed by a high temperature and a rapid pulse. Vomiting supervened and the patient died upon the thirteenth day. The value of milk as a food in typhoid fever was then discussed.

Dr. Wood thought it a case in which milk was injurious, and he considered the feeding of milk a frequent mistake. Some patients have idiosyncrasies that make milk harmful. Again, it is a proper food late, but not early, in the disease. Good digestion of milk is often insured when whey or half milk and whey is given.

Dr. J. M. Batten thought the case a malignant one and its termination in no manner influenced by the milk administered. The temperature should have been controlled by cold baths instead of by quinine, as was done.

Dr. W. D. Kearns said beef-tea is proper food when milk is rejected, and that high temperature is not to be feared to the extent formerly believed. He never exhibited antipyretics in his practice, but stimulated the patient, giving quinine and carbonate of ammonia.

Dr. Stewart called attention to the utility of buttermilk where milk was rejected.

Dr. McCann thought as a rule typhoid fever is badly treated. The patient is over-medicated and over-fed. Milk, because of its decomposition in the intestinal canal, is not a safe food. During this process the poisonous animal alkaloids are formed, and they, acting through the nervous system, induce relapses, high temperatures and other unfavorable events. He uses antifebrine and sponge baths for high temperature and prefers gruels, vegetable juices and beef-tea to milk.

Dr. E. T. Painter read a paper on

A CASE OF ŒSOPHAGEAL STRICTURE.

Dr. Duff presented a case of purpura simplex.

A child had been suffering from diarrhœa for some three or four weeks and had received home remedies which would check it for a time, but it had returned in a few days. The child had been in this condition until the time I saw it. Upon seeing it I discovered at once it was a case of what I would term purpura simplex. There were spots of all grades upon its back and extremities, some very livid and some a pale, yellow cover as they were disappearing, all being, with one exception, about the size of a finger nail; one on the back was perhaps the size of a silver dollar. I gave the child some bismuth and pepsin, and in the *interim* between the doses of pepsin I had it take two drops of aromatic sulphuric acid. It improved in a few days and then took a slight relapse, so far as the diarrhœa was concerned, but the spots continued to disappear. It got so well that I didn't see it for several days. I was sent for perhaps a week afterwards and notified that the diarrhœa had returned with vomiting. I saw it and continued the same treatment with the addition of lime water. The child improved again for three or four days, when I discharged it, as I thought it comparatively well. Its mother took sick within a few days after this and I attended her for two or three days, when my attention was drawn to the child as suffering very materially from its teeth, as it was biting its gums and crying a great deal. I examined its teeth, or its gums, and found them very much swollen. In the region of the upper canine teeth I examined particularly. I thought to relieve it by scoring the gum; this was in the evening about five or six o'clock. I scored over one of the canine teeth and went home thinking nothing of it. About daylight I was called to the house, and found the child lying in bed, the mother's clothing all over blood, and the bed bloody and the child pulseless. I administered restoratives and worked with it for sometime until it revived somewhat. In the afternoon I saw it again and the purpuric spots had reappeared on its back, and there was some hemorrhage from the bowels as well as hemorrhage from the bladder. The child died the next morning. I may say that it is not strictly a case of hæmophilia. I examined the histories of the father's and mother's side, and there was no history of hemorrhage in the family, with the exception of the father, who has frequently had severe hemorrhages from the nose. There is no history of tubercular trouble in either branch.

Dr. J. J. Buchanan presented a paper on

### FRACTURED PATELLA TREATED BY WIRING.

Dr. Murdoch said the case is certainly a very interesting one, and, as far as I know, it is the first case where the patella has been wired, either for simple or compound fracture, in this city. Every one who has had much practice in fractures of the patella has found some difficulty in keeping the fragments in position. Long before the use of antisepsis, or any attempt thereat, in order to hold the bones in position, Malgaigne's hooks were used— to be had yet, I suppose, in any of our surgical instrument stores.

They were used in such cases successfully, but owing to the fact, probably, that the wound was not made aseptic, the operation was abandoned as leading to inflammation and disease in the joint.

The operation for union of the patella is one which the doctor says has increased risk. By the old method of treating fractures of the knee, the patient usually got well. For my own part, I think this operation involves a great risk, which a surgeon ought to estimate at its just weight. There have been several deaths from wiring the patella. In all cases of compound fracture of the patella, it should be wired if there is difficulty in keeping the fragments together by the ordinary apparatus.

I would say that, perhaps, in some cases it should be wired by a surgeon who is a master of antisepsis, and to those who have not the facilities of carrying it out thoroughly I would say, be careful; you might sacrifice a life; whereas, by using the old method of treatment, you would be perfectly safe, and would save the patient, possibly, with a halt in the gait. In one thing I disagree with Dr. Buchanan, and that is the idea of leaving such a serious matter to the patient himself. I don't think any patient is capable of judging the dangers he runs in undergoing an operation of that kind. He knows nothing about the value of antiseptics in the treatment of wounds; he doesn't know that it is any different to perform an operation with antiseptic treatment than without; and to leave the question to a man ignorant of such matters, without being able to tell him how much risk he runs, I do not think it proper or right. I think the surgeon in such a case should be the judge, and should say to the patient which treatment will be proper.

Dr. Buchanan : I would like to say that the patient ought to have the first choice. If he should say, "I consider my life of the first importance; I don't want it to be put in the slightest jeopardy," as any of you gentlemen might say, then I take it to be the surgeon's duty to say, "We'll do the best we can for you." But as this man says, "I am a laborer; my life depends on the usefulness of my limb; if I have a crippled limb I cannot make my living; how much risk is there?" I say to him, "I don't think there is one chance out of ten, probably not one chance out of twenty, that you will die." And there are cases by the dozen where the conditions are as I have stated, and where the patient will say, "I will take that chance; give me the strongest limb, for I can't make my living without it." Under these circumstances, I think that the man has a right to his choice, and I think we can give him some idea of the risk he runs by telling him how many in the long-run lose their lives by the operation.

---

A VALUABLE REMEDY FOR CHRONIC DIARRHŒA.—I think you would do a service if you would call attention in your journal to a valuable remedy for chronic diarrhœa.

Many years ago I suffered severely from that trouble; I considered it incurable. Being in Paris, one of the best physicians there assured me it could be cured by a diet of racahout, and it was.

Afterward here I found one could not get the acorn meal that forms the active part, but knowing that its usefulness must depend on the tannin it contains, I tried substituting it as follows:

| | |
|---|---|
| Powdered chocolate, pure...... | ½ lb. |
| Rice flour...................... | ½ " |
| Powdered sugar............... | ½ " |
| Tannin......................... | ¼ oz. (120 grs.) |

The tannin or the rest, separately, have little effect. Together they restore the tone of the alimentary canal and nourish as well as cure.

One thing is *essential*, that is long cooking, not less than half an hour. If simply boiled a few minutes the harsh taste of the tannin is very strong; with a good half hour's cooking it disappears *wholly*—it is impossible to distinguish the medicine from ordinary broma. I think this has something to do with its curative powers and with the ease of digestion by the most irritable stomach. The remedy is too valuable not to be more widely known.

The amount to be taken is a heaping table spoonful cooked, as directed, in a cup of milk morning and evening at meals.—*Medical News.*

